# Wild mushroom poisoning in Mengzi city, Yunnan province, China, 2018–2023: an analysis through epidemiological characteristics and time series analysis with SARIMA and holt-winters models

**DOI:** 10.3389/ftox.2025.1705460

**Published:** 2025-12-05

**Authors:** Rong Niu, Yimin Wang

**Affiliations:** School of Public Health, Lanzhou University, Lanzhou, Gansu, China

**Keywords:** wild mushroom Poisoning, epidemiological characteristics, time series analysis, SARIMA model, Holt-Winters model

## 1 Introduction

Mushrooms are a mostly edible group of macroscopic fleshy fungi that grow from mycelia. As of 2017, approximately 150,000 fungal species have been scientifically described worldwide, representing only 4%–7% of the estimated total (Hawksworth and Lücking, 2017). There are about 17,000 species in China alone (Yang et al., 2024). Among them, 1020 are edible and 660 are toxic. China is the country with the largest number of known toxic species. Wild mushroom poisoning is an important global foodborne disease, mainly mediated by structurally determined mycotoxins. According to different mechanisms of action, these toxins can be divided into 7 categories: Amatoxins blocks protein synthesis by inhibiting RNA polymerase, causing necrosis of liver and kidney cells; Orellanines accumulates in the kidney, destroys renal tubular epithelial cells, causing irreversible kidney damage; Psilocybin acts on the central 5-serotonin receptor after transformation in the body, interfering with nerve signal transmission and induces hallucinations; the methyl methylene produced by Gyromitrin not only inhibits the destruction of liver enzyme to destroy liver cells, but also damages the nervous system and may cause hemolysis; Muscarine activates peripheral cholinergic M receptors, causing increased glandular secretion, gastrointestinal spasms and pupil narrowing; Ibotenic acid and Muscimol first excites the central center and then inhibits nerve activity to cause lethargy coma; Hemolysins directly destroys the red blood cell membrane, leading to anemia and jaundice (Wennig et al., 2020). The morphological similarity between toxic and edible species, combined with limited public awareness, makes the high incidence of such poisoning persist for a long time.

In Iran alone, 1,247 cases of poisoning were reported in 2018, resulting in 112 hospitalizations (8.9%) and 19 deaths, with a death rate of 1.5% (Soltaninejad, 2018). In China, the long tradition of picking and eating wild fungi causes many poisoning cases every year. Due to its unique climate and geographical location, Yunnan Province has always been the province with the highest incidence rate in the country. Mengzi City is located in the southeast of Yunnan, the capital of Honghe Hani and Yi Autonomous Prefecture. Its low-latitude plateau subtropical monsoon climate provides uniquely favorable conditions for wild mushroom growth. Sufficient rainfall and suitable temperature together create a continuous humid environment, and the local acidic and fertile soil provides an ideal substrate for the spread of mycelium, which significantly promotes the growth and reproduction of fungi. Diverse microtopography and obvious wet-dry season alternate to form a unique growth cycle, making mushrooms a valuable ecological and seasonal resource. In recent years, the epidemiological profile of wild mushroom poisoning in the region has shown obvious laws and evolutionary trends.

Although there is a serious risk of mushroom poisoning, existing studies are largely limited to retrospective descriptive analysis. Poisoning incidents have obvious seasonal and annual cycles. Although models such as Prophet or machine learning methods are suitable for complex multivariate scenarios, SARIMA can effectively capture autocorrelation, seasonality, and trend components in a single-variable time series (Taylor and Letham, 2018). The Holt-Winters model is suitable for short-term forecasts with obvious seasonal characteristics but relatively stable trends. Therefore, classical statistical models—which have been well verified in epidemiological prediction, are highly interpretable, and suitable for single-variable time series—are prioritized to provide explainable predictions while ensuring robustness. This study aims to establish SARIMA and Holt-Winters exponential smoothing models to predict future incidence trends. Based on the model results, prevention and control suggestions are put forward.

## 2 Methods

### 2.1 Dataset

**FIGURE 1:**
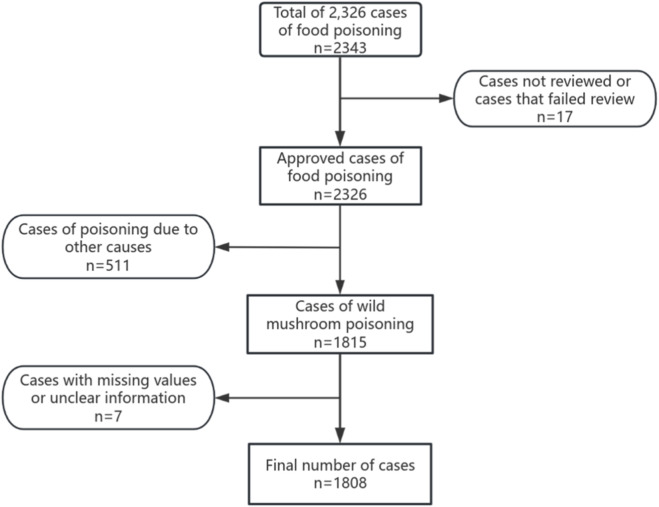
Case inclusion flowchart.

This data comes from the “Foodborne Disease Case Monitoring Report System”, which reports foodborne disease cases in Mengzi City from 2018 to 2023. Data that has not been approved, there are missing values, insufficient evidence, or vague information is excluded. According to the exposure history, clinical symptoms and diagnostic results of wild mushrooms, the cases diagnosed with wild mushroom poisoning were included in the analysis. The case inclusion flowchart is shown in Figure 1.

### 2.2 Diagnostic methods

Diagnostic criteria for wild mushroom poisoning: 1. Epidemiological investigation evidence, including the history of eating wild mushrooms and wild mushroom samples collected on the spot; 2. Clinical manifestations of patients, such as poisoning symptoms; 3. Laboratory test results, including morphology, toxin detection, and gene sequencing of wild mushroom samples.

### 2.3 ARIMA model

The basic expression is ARIMA (p, d, q) × (P, D, Q) s, where both non-seasonal and seasonal components must be specified (Tobias et al., 2001). Model development follows a structured process: First, assess the initial stationarity of the time series, applying differencing (d, D) if necessary. Subsequently, identify potential (p, q) and (P, Q) orders by analyzing the autocorrelation function (ACF) and partial autocorrelation function (PACF) plots of the differenced series. Model selection rejects vague heuristics by systematically comparing the Akaike Information Criterion (AIC) and Bayesian Information Criterion (BIC) of candidate models, prioritizing those with lower values (Wang et al., 2018). Diagnostic validation ultimately ensures the selected model’s residuals exhibit white noise characteristics, typically confirmed using the Ljung-Box test (Xia et al., 2020).

### 2.4 Holt-winters exponential smoothing

The method includes three parameters, α, β, and γ, which control the weight adjustments for the level, trend, and seasonal components, respectively (Chatfield, 1978). The parameter value ranges from 0 to 1, and the closer to 1, the greater the impact of recent data on the prediction. To overcome subjective manual specification, optimal parameter combinations are systematically identified through grid search methods that minimize objective functions such as the AIC, thereby enabling data-driven model configuration and enhancing forecasting accuracy (Chatfield, 1978).

### 2.5 Statistical analysis

Data were organized and analyzed using SPSS 23.0 software. The Mann-Kendall test was employed to assess trends in case numbers from 2018 to 2023. Chi-square tests were performed for gender distribution, age distribution, occupational distribution, time of onset distribution, symptom distribution, and exposure food sources and locations. In this study, p < 0.05 was considered statistically significant. Concurrently, R4.4.3 was used to construct SARIMA models and Holt-Winters exponential smoothing models to predict the future wild mushroom poisoning situation in Mengzi City.

## 3 Results

### 3.1 General information

From 2018 to 2023, Mengzi City reported a total of 2,326 cases of food poisoning. Among them, 1,808 cases were caused by wild mushrooms, including 59 hospitalizations, 1,749 outpatient cases, and 2 deaths, with a standardized incidence rate of 46.09/100,000. The annual average incidence was 301 cases. Cases of hospitalization due to wild mushroom poisoning accounted for 56.73% (59/104) of all food poisoning hospitalizations in the city.

At the provincial level, Yunnan Province reported 9,686 wild mushroom poisoning cases from 2010 to 2018, including 2,030 cases in 2018 alone (Jiang et al., 2019). That year, Mengzi City reported 231 cases, accounting for 11.38% of the provincial total. Compared with other cities and prefectures in Yunnan Province, the wild mushroom poisoning situation in Mengzi City is not as severe as in Chuxiong Prefecture and Lijiang, but it is still relatively serious compared to some other cities and prefectures (Table 1).

**TABLE 1:** Wild mushroom poisoning in individual areas of Yunnan province.

Region	Cases/Year	Year
Yunnan	1076.2	2010–2018
Chuxiong Prefecture (Mei-qiong et al., 2019)	602	2012–2018
Lijiang City (Yong-li et al., 2022)	347.42	2014–2020
Baoshan City (Jia-Yan and Wei-Bin, 2019)	81	2010–2018
Dali Prefecture (Shunzhu, 2019)	92	2010–2018

### 3.2 Epidemiological characteristics

#### 3.2.1 Temporal distribution

From 2018 to 2023, there was a significant linear decline trend in the number of wild mushroom poisoning cases in Mengzi City (z = −23.128, P < 0.01). The number of cases increases significantly from 2018 to 2019, peaking in 2019 (20.80%), whereas hospitalized cases peaked in 2022 (22.03%) (Table 2).

**TABLE 2:** Annual monitoring of wild mushroom poisoning in Mengzi city from 2018 to 2023.

Year	Residents (ten thousand)	Cases	Hospitalizations	Deaths
		Number	Number	Number
2018	45.83	231 (12.78)	4 (6.78)	0
2019	50.40	376 (20.80)	11 (18.64)	1 (50.00)
2020	58.59	364 (20.13)	11 (18.64)	1 (50.00)
2021	59.03	293 (16.21)	12 (20.34)	0
2022	59.33	290 (16.04)	13 (22.03)	0
2023	59.51	254 (14.05)	8 (13.56)	0
Total	​	1808	59	2

**FIGURE 2:**
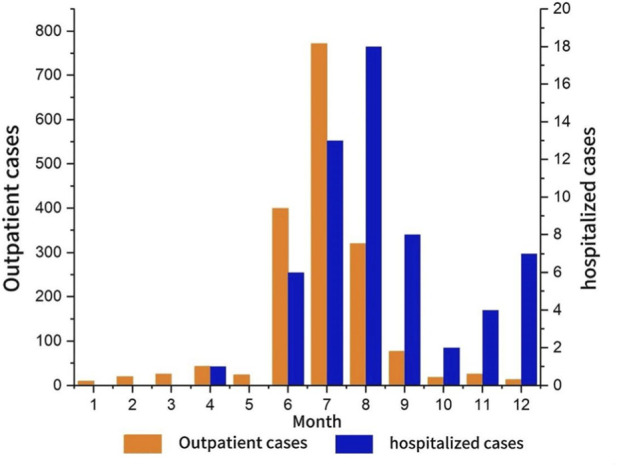
Monthly monitoring of wild mushroom poisoning in Mengzi city note: produced based on the Mengzi city map with review number Yun S (2024)114 downloaded from the Yunnan provincial geographic information public service platform.

Although cases occurred year-round, wild mushroom poisoning showed clear seasonality. Most cases were reported between June and August, accounting for 84.57% (1529/1808) of the total. Both reported deaths also occurred during these peak months. July had the highest number of cases, contributing 42.42% (785/1,808), making it the month of greatest risk. However, August had the highest number of hospitalizations, accounting for 30.51% (18/59) (Figure 2).

#### 3.2.2 Regional distribution

**FIGURE 3:**
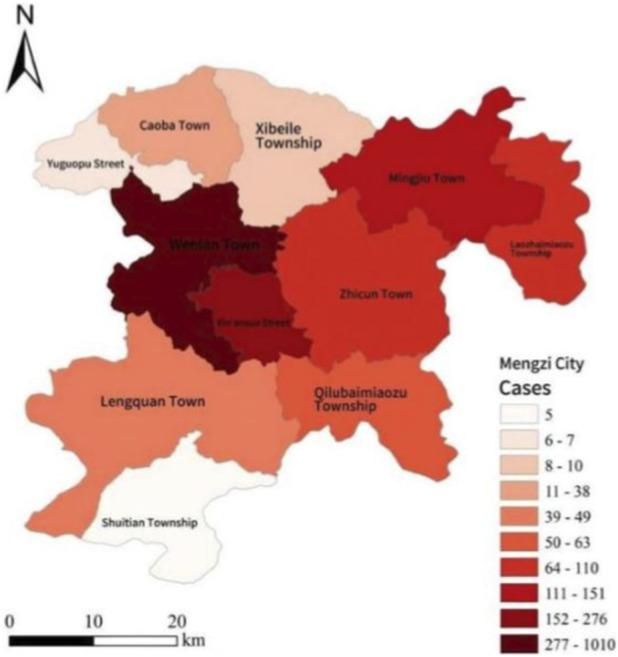
Distribution of areas with wild mushroom poisoning in Mengzi city.

Among the 11 subordinate areas of Mengzi City, Wenlan Town reported the highest number of wild mushroom poisoning cases and hospitalizations. It accounted for 55.86% (1,010/1,808) of total cases and 63.60% (40/59) of total hospitalizations. In contrast, Shuitian Township reported the fewest cases, accounting for only 0.28% (5/1,808). The regional differences in the number of cases were statistically significant (χ^2^ (10, N = 1808) = 4997.04, P < 0.001) (Figure 3).

#### 3.2.3 Population distribution

In terms of gender, the male-to-female ratio was relatively balanced. The highest number of cases and hospitalizations occurred in the 35–64 age group, with 1,077 cases (59.57%) and 27 hospitalizations (45.76%). The two deaths were reported in the 35–64 and ≥65 age groups. Regarding occupation, farmers were the most affected group, accounting for 57.36% (1,037/1,808) of total cases. Both deaths occurred in male farmers (Table 3).

**TABLE 3:** Distribution and analysis of suspected exposure foods in wild mushroom poisoning cases in mengzi city from 2018 to 2023.

Category	Cases		Hospitalizations		Deaths	
	Number	%	Number	%	Number	%
Gender						
Male	933	51.60	35	59.32	2	100.00
Female	875	48.40	24	40.70	0	0.00
Age						
0–5	12	0.66	3	5.08	0	0.00
6–12	60	3.32	8	13.56	0	0.00
13–17	67	3.71	1	1.69	0	0.00
18–34	442	24.45	7	11.86	0	0.00
35–64	1077	59.57	27	45.76	1	50.00
65–94	150	8.30	13	22.03	1	50.00
Occupation						
Farmer	1037	57.36	26	44.07	2	100.00
Catering and food industry	3	0.17	0	0.00	0	0.00
Cadre	57	3.15	2	3.39	0	0.00
Worker	95	5.25	2	3.39	0	0.00
Teacher	17	0.94	0	0.00	0	0.00
Herdsmen	2	0.11	1	1.69	0	0.00
Scattered children	16	0.88	5	8.47	0	0.00
Commercial service	9	0.50	0	0.00	0	0.00
Nursery children	9	0.50	2	3.39	0	0.00
Medical personnel	6	0.33	0	0.00	0	0.00
Students	115	6.36	5	8.47	0	0.00
Retired	92	5.09	13	22.03	0	0.00
Housework and Unemployed	70	3.87	1	1.69	0	0.00
Other	280	15.49	2	3.39	0	0.00
Dining venue						
Catering Services	44	2.43	0	0.00	0	0.00
Collective Canteen	45	2.49	0	0.00	0	0.00
Family	1714	94.80	59	100.00	2	100.0
Other	5	0.28	0	0.00	0	0.00
Processing and packaging method						
Catering Services	44	2.43	0	0.00	0	0.00
Homemade	1744	96.46	57	96.61	2	100.0
Bulk (including simple packaging)	6	0.33	2	3.39	0	0.00
Other	14	0.77	0	0.00	0	0.00
Suspected exposure food source distribution						
Catering Services	48	2.65	1	1.69	0	0.00
Collective Canteen	1	0.06	0	0.00	0	0.00
Family	373	20.63	15	25.42	1	50.00
Street Food	11	0.61	0	0.00	0	0.00
Retail Market	977	54.04	36	61.02	0	0.00
Other	398	22.01	7	11.86	1	50.00

#### 3.2.4 Symptom distribution

All 1,808 cases reported clinical symptoms, with the majority presenting multiple symptoms per case. The symptom distribution showed significant patterns, with the digestive system being the most affected, accounting for 73.30% of all symptoms. Among these, nausea accounted for 82.90% (1457/1721), vomiting 80.38% (1438/1721), abdominal pain 40.88% (662/1721), and diarrhea 36.82% (586/1721) (Table 4). Vomiting typically occurred 3 to 10 times per day. Diarrhea was mainly watery, followed by loose stools. Respiratory symptoms were rare, accounting for only 0.05% of all symptoms.

**TABLE 4:** Distribution of symptoms of wild mushroom poisoning in Mengzi City from 2018 to 2023.

Symptoms	Number	%
Systemic symptoms and signs	315	14.33
Digestive system	1721	78.30
Respiratory system	1	0.05
Cardiovascular and cerebrovascular system	15	0.68
Urinary system	2	0.09
Nervous system	141	6.41
Skin and subcutaneous tissue	3	0.14

#### 3.2.5 Analysis of suspected exposed food

The dining venues involved in the mushroom poisoning incidents in Mengzi City have different characteristics. Among the 1,808 cases, 1,714 occurred in households, accounting for 94.80% of all cases. The hospitalization rate for these household cases was high, and both deaths occurred in home settings. A total of 1,744 cases involved home-based processing, accounting for 96.46% of all cases, with 96.61% (57/59) of hospitalizations also associated with home processing. Regarding food exposure sources, the retail market was the main source, accounting for 54.04% (977/1808) of the total cases. Home collection was the second most common source, at 20.63% (373/1,808) (Table 4). There were statistically significant differences (P < 0.001) in the number of mushroom poisoning cases across different dining venues, processing and packaging methods, and food exposure sources.

### 3.3 Time series analysis

#### 3.3.1 SARIMA model

**FIGURE 4:**
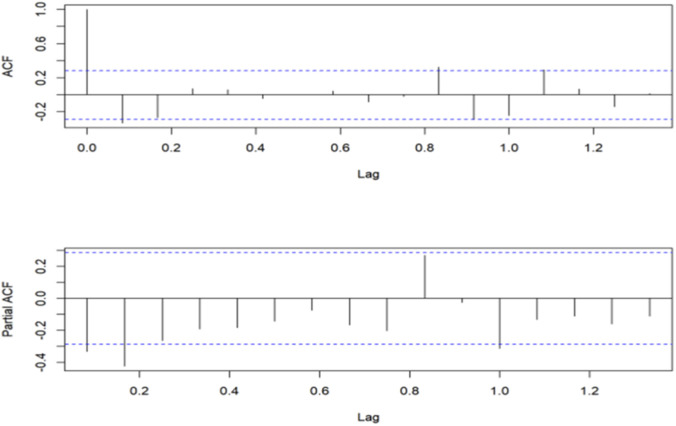
ACF and PACF diagram after differencing.

**FIGURE 5:**
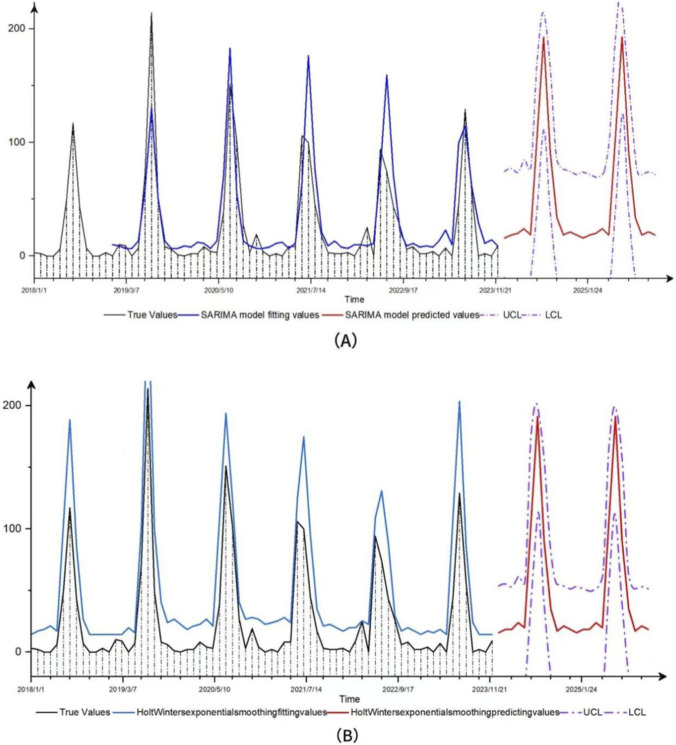
Fitting and prediction of two models for wild mushroom poisoning in Mengzi city. **(A)** SARIMA model. **(B)** Holt-Winters exponential smoothing model.

The number of wild mushroom poisoning cases in Mengzi City from 2018 to 2023 showed a downward trend, indicating a non-stationary time series. After applying first-order seasonal differencing (D = 1), the data satisfied the stationarity condition. The autocorrelation function (ACF) decayed gradually (q ≥ 1), while the partial autocorrelation function (PACF) truncated after lag 1 (p ≤ 1) (Figure 4). According to the empirical rule that seasonal parameters P and Q usually do not exceed 2, the optimal model was identified as SARIMA (1,0,0) (0,1,1)_12_through parameter screening. The Box-Ljung test indicated that the residuals of the fitted model constituted a white-noise series (p > 0.05), confirming the absence of significant autocorrelation. Together with a high *R* ^2^ value of 0.816, these results indicate that the model had a good fit (Figure 5). Therefore, we employed this model to predict the number of wild mushroom poisoning cases in Mengzi City during 2024–2025. The forecast indicates a slight increase, with 296 cases expected over the next 2 years.

#### 3.3.2 Holt-winters exponential smoothing model

Because the data from 2018 to 2023 exhibit a fixed seasonal pattern, the Holt-Winters additive model was also applied for comparison (AIC = 630.28, RMSE = 18.575, MAE = 10.97). This model predicted 295 cases for both 2024 and 2025, also indicating a slight rise.

#### 3.3.3 Model evaluation

The fitting effects of the two models are compared through parameters. The results show that, compared with the Holt-Winters additive model, SARIMA (1, 0, 0) (0, 1, 1)_12_ has lower values for the two indicators, MAE and MASE, and a higher R value, Therefore, SARIMA (1,0,0) (0,1,1)_12 _model has the best fitting and prediction effects (Table 5).

**TABLE 5:** Comparison of 2 model parameters.

Model	Box-ljung test (*P*)	AIC	R	MAE	RMSE	MASE
SARIMA (1,0,0) (0,1,1)_12_	0.8226	454.67	0.816	10.13	20.53	0.40
Holt-Winters Additive Model	0.7152	223.14	0.710	10.97	18.57	0.76

## 4 Discussion

This study found that from 2018 to 2023, Mengzi City reported a total of 1,808 cases of wild mushroom poisoning, including 1,749 outpatient cases, 59 hospitalizations, and 2 deaths, with an average annual number of 301 cases. The case fatality rate remained at a low level.

The severity of the wild mushroom poisoning incident in Mengzi City deserves attention at home and abroad (Table 6). In addition to countries with high consumption of wild mushrooms such as Russia and Eastern Europe, China also reports a large number of poisoning cases, and significant differences have been observed between different provinces. China, like Russia and Ukraine, belongs to the regions with higher global case fatality rates of wild mushroom poisoning. Among Asian countries, China has the highest case fatality rate. In 2003, Guangdong Province recorded 6 wild mushroom poisoning events, leading to 33 affected individuals and 20 deaths (Sun et al., 2018). In 2018, Mengzi City reported 231 wild mushroom poisoning cases, accounting for 11.38% of Yunnan Province’s total that year. Although lower than in Chuxiong and Lijiang, this number was still higher than in most other areas of the province. Moreover, Mengzi City’s standardized incidence rate (46.09/100,000) is higher than that of most regions.

**TABLE 6:** Global wild mushroom poisoning.

Region	Number of cases	Cases/year	Deaths	Deaths/year	Year
United States (Brandenburg and Ward, 2018)	133700	7428	52	2.9	1999–2016
Turkey (Turan Gökçe et al., 2024)	30459	5076.5	205	41	2018–2023
United Kingdom (Edwards et al., 2025)	1195	119.5	​	​	2013–2022
Thailand (Somrithipol et al., 2022)	22571	1504.73	106	7.07	2003–2017
Iran (Soltaninejad, 2018)	1247	​	19	​	April 28 to 28 May 2018
Mazandaran, Iran (Khatir et al., 2020)	65	16.25	0	​	2015–2018
Russia (Govorushko et al., 2019)	​	806.5	​	260	2005,2010,2013–2016
Munich (Wennig et al., 2020)	6374	1062	​	​	2018–2023
Japan (Govorushko et al., 2019)	1920	192	10	1	2001–2010
Israel (Lewinsohn et al., 2023)	105	105	​	​	2020
	614	61.4	​	​	2010–2020
Apulian (Pennisi et al., 2020)	69	69	​	​	2018
Southern Sardinia (La Rosa et al., 2024)	164	16.4	​	​	2011–2021
Switzerland (Schmutz et al., 2018)	87	8.7	​	​	2004–2014
China (Li et al., 2021)	38676	3516	​	​	2010–2020
China (Guizhou) (Zhang et al., 2023)	5312	482.91	95	8.64	2011–2021
China (Zhejiang) (Chen et al., 2022)	429	143	2	0.67	2016–2018
China (Jiangxi) (Xingyong et al., 2019)	463	77.17	19	3.17	2012–2017

The number of cases of wild mushroom poisoning in Mengzi City showed a significant linear downward trend in 6 years. However, from 2018 to 2019, this figure rose sharply. This change may be closely related to the full coverage of the foodborne disease monitoring network in Yunnan Province in 2018. June to August is the peak of fungal growth, which is consistent with the growth conditions and feed structure of fungi (Wenli et al., 2021). This seasonal model is consistent with the survey results of Yunnan Province (Xiu-lian et al., 2022) and Guizhou Province (2015–2017) (Ya-fang et al., 2019).

The poisoned population is mainly concentrated in the urban area of Mengzi City. The higher population density and greater demand for wild mushrooms in urban areas, coupled with complex supply chains such as markets and mobile vendors, make regulation difficult. In terms of the age and occupational distribution of the poisoned population, it is mainly concentrated in the farmers aged 35–64. This group is generally less educated and has limited access to health and safety information. Moreover, many farmers lack stable living or working conditions and are more likely to purchase food from mobile sources. These behaviors increase the risk of accidental poisoning. In addition, rural and urban residents generally lack knowledge of how to identify poisonous mushrooms. Therefore, it is necessary to strengthen market inspections, block the circulation of poisonous fungi, and strengthen public education on the safety of wild mushrooms, especially among mobile populations. In terms of edible and processing places, households are the main high-risk place for wild mushroom poisoning in Mengzi City. This discovery is consistent with data of Yunnan Province (Xiu-lian et al., 2022) and Guizhou Province (Ya-fang et al., 2019). Most of the poisoning cases involve mushrooms from the retail market. There is a designated wild mushroom trading area in Mengzi City. Wild mushrooms are purchased in bulk from surrounding townships or sold directly by villagers. This further explains the possible reason for the high incidence of poisoning in urban areas. Despite the efforts of regulators to crack down on illegal traders, persistent informal trade continues to challenge market regulation. It is recommended to strengthen market control and targeted public education to reduce the incidence of poisoning. In addition, public education activities should be carried out to improve residents’ ability to identify toxic species and raise awareness of food safety, thus reducing the risk of accidental ingestion at its source.

The diagnosis and treatment of wild mushroom poisoning mainly rely on clinical symptoms, exposure history, incubation period and other factors. The toxic dynamics of different toxins determine the length of their incubation period, the type of symptoms and the severity. Clinical manifestations can range from self-limiting gastrointestinal irritation to specific organ failure and even death (Karlson-Stiber and Persson, 2003). The toxicity of mushrooms stems from the interaction between their biosynthetic products and the external environment, which is highly complex. Toxicity intensity is not only species-specific but also influenced by multiple factors such as the maturity of the cotyldon, the harvest season, the geographical source and the most important cooking method. Take the common varieties in the market of Mengzi County as an example, such as the boletus edulis, other bolete species, lactarius mushrooms, blue-capped mushrooms, dryad’s saddle mushrooms, and red mushrooms. Many unprocessed mushrooms themselves contain trace amounts of natural toxins. Improper pretreatment or incomplete cooking is easy to lead to toxin intake and poisoning.

From the perspective of toxicological syndrome, the distribution of cases in Mengzi City closely related to the mechanism of mushroom toxins. Most cases present as acute gastrointestinal syndrome, rooted in the toxins’ direct irritation of the gastrointestinal mucosa. The incubation period is short (0.5–4 h), and the symptoms are serious, but it is usually self-limiting (Karlson-Stiber and Persson, 2003). It is worth noting that about 25% of the cases described in this article can be attributed to improper consumption of boletus. This type of poisoning is characterized by neuropsychiatric syndrome. The neurotoxins in undercooked false morels act on the central nervous system, causing hallucinations, delirium, and other psychiatric symptoms.

However, it must be emphasized that hepatotoxic syndrome has the greatest toxicological significance among all types of poisoning. Although it may represent a smaller proportion of cases in Mengzi County, the incubation period of poisoning caused by amatoxins is longer (6–24 h). Its mechanism involves the irreversible inhibition of liver RNA polymerase by toxins, which leads to the cessation of protein synthesis and acute liver necrosis, resulting in a very high mortality rate (Wennig et al., 2020).

Among the 1,808 cases reported in Mengzi City, vomiting, diarrhea and other gastrointestinal symptoms were common, and most of the patients received outpatient treatment. This precisely confirms that the gastrointestinal syndrome represents the most common yet relatively mild form of poisoning in the region.

Foodborne poisoning often results in a substantial disease burden. Timely prediction and control can help reduce these losses. Among the many prediction models, the SARIMA model is widely used for foodborne diseases due to its ability to capture trends, seasonality, and random fluctuations in time series data (Mingwen et al., 2021). For example (Zi-yang et al., 2021), applied a multiplicative seasonal ARIMA model to predict the monthly incidence of foodborne diseases in Yunnan Province. However, SARIMA model requires stricter data stationarity. In contrast, the Holt-Winters exponential smoothing model has lower stationarity requirements. It is more suitable for time series with strong trends and seasonal but non-stationary patterns.

In this study, both the SARIMA model and the Holt-Winters exponential smoothing model showed good predictive ability for the number of wild mushroom poisonings. According to model forecasts and field observations, it is expected that the number of cases of wild mushroom poisoning will increase from 2024 to 2025. This growth is mainly due to the increasing complexity of regulatory sales channels (including online and offline) and the continuous improvement of the foodborne disease monitoring system. In the future, the two models can be combined to improve the prediction accuracy through complementary advantages.

This study has certain limitations. The prediction model did not take into account important external factors. As we all know, wild mushroom poisoning is affected by weather conditions such as rainfall and temperature, market control and public awareness. In addition, there may be errors in the reporting system of poisoning cases. Some cases may not be reported, and some reports may be wrong. Future research should optimize or reconstruct the model in combination with newly collected multi-source data (Jian-li et al., 2013), and integrate key variables such as meteorological factors, market supervision intensity, and health education coverage to establish a more accurate poisoning risk prediction framework.

In summary, despite the overall decline in the report of wild mushroom poisoning in Mengzi City in 2018–2023, the situation is still serious and public health intervention needs to be strengthened. In order to further reduce the incidence of poisoning, we suggest: (1) strengthen seasonal early warning and targeted health education in high-risk months; (2) establish a rapid response network connecting communities, clinics and disease control departments to improve early diagnosis and management; (3) establish a comprehensive forecasting model. The SARIMA model performed well in this study. Combined with additional factors such as meteorological and mobile data, it can improve the accuracy of its prediction of the risk of poisoning, thus supporting targeted prevention strategies. Research shows that the SARIMA model has good predictive performance. In the future, we can further integrate meteorology, vegetation, crowd flow and other influencing factors, build a more accurate poisoning risk early warning system, and provide a scientific basis for the implementation of targeted prevention and control.

## Data Availability

The raw data supporting the conclusions of this article will be made available by the authors, without undue reservation.
